# Selecting Suitable Nitrogen Offset Strategies In Tropical And Subtropical Regions Globally With Implications To The Great Barrier Reef, Australia

**DOI:** 10.1007/s00267-026-02466-5

**Published:** 2026-04-30

**Authors:** Diego F. Correa, Ryan D. R. Turner, Martine Maron, Lina M. González-González, Michele A. Burford, Jing Lu, Joanne Burton, Joseph McMahon, Debra Chamberlain, Yongjing Mao, Hugh Possingham, Michael St. J. Warne

**Affiliations:** 1 Reef Catchments Science Partnership RCSP, School of the Environment, The University of Queensland, Brisbane, 4067 QLD Australia; 2 Centre for Biodiversity and Conservation Science CBCS, The University of Queensland, Brisbane, 4067 QLD Australia; 3 Water Quality and Investigations, Queensland Department of Environment and Sciences, Brisbane, QLD Australia; 4 School of the Environment, The University of Queensland, Brisbane, 4067 QLD Australia; 5 The Bashan Institute of Science, 1730 Post Oak Ct, Auburn, AL USA; 6 Australian Rivers Institute, Griffith University, Nathan Campus, Brisbane, 4111 QLD Australia; 7 Council of Mayors South East Queensland, Brisbane, 4003 QLD Australia; 8 School of Civil and Environmental Engineering, University of New South Wales, Sydney, 2052 NSW Australia; 9 Centre for Agroecology, Water and Resilience, Coventry University, Coventry, UK

**Keywords:** Nitrogen offsets, Water quality management, Performance assessment, Efficiency and effectiveness, Watershed resilience

## Abstract

Water pollution from agricultural, industrial, and urban activities threatens aquatic ecosystems and the essential services they provide. Excess nitrogen has been identified as a key driver of water quality degradation, impacting biodiversity, food security, and human health. One approach to mitigating nitrogen pollution is water quality offsetting, compensating for pollution impacts by implementing nitrogen reduction measures elsewhere to achieve no net decline in water quality. Selecting and implementing suitable nitrogen offset types remains challenging. This study presents a framework for identifying, selecting, and implementing effective nitrogen offset strategies in tropical and subtropical regions globally, using the Great Barrier Reef Catchment Area (GBRCA) as an example. This framework is based on the premise that effective nitrogen offset design requires integrating evidence on nitrogen mitigation performance, appropriate criteria for selecting offset types, and adaptive management to address uncertainty in environmental outcomes. Accordingly, the framework comprises three interrelated components: (1) assessment of water quality improvement methods for nitrogen reduction in the GBRCA and other tropical and subtropical regions based on performance (i.e., efficacy, effectiveness, and efficiency); (2) selection of suitable offset types based on their performance, co-benefits, and spatial and technical feasibility; and (3) integration of adaptive management strategies, Geographic Information Systems (GIS) tools, and monitoring systems to strengthen the effectiveness of nitrogen offsetting. This is the first global assessment of nitrogen offset performance in tropical and subtropical regions, offering insights to improve water quality management through offsetting. This framework is adaptable to other regions and pollutants, guiding effective offset implementation to enhance watershed resilience.

## Introduction

Water pollution from agricultural, industrial, and urban development negatively impacts aquatic ecosystems (Smith 2003; UNESCO 2021; Vorosmarty et al. 2010) and the multiple services they provide (MEA 2005; UN 2016). Unless environmentally sound solutions for reducing water pollution are implemented (UN 2018), a considerable proportion of the global population and ecosystems will likely suffer the impacts of water degradation (Boretti and Rosa 2019), including declines in human health (Schwarzenbach et al. 2010), food security (FAO 2020; Rosegrant et al. 2009), and biodiversity (MEA 2005; UN 2016). In particular, Goal 6 of the United Nations Sustainable Development Goals (SDGs) aims to ensure the availability and sustainable management of water and sanitation globally by eliminating or minimizing the release of pollutants into water bodies (UN 2015, 2022). Excess nitrogen has been identified as a major pollutant in aquatic ecosystems, where it disrupts natural nutrient balances and drives eutrophication (Ghaly and Ramakrishnan 2015; Howarth 2008). Eutrophication promotes the rapid growth of algae, several of which are toxic, leading to hypoxic or anoxic conditions (Glibert et al. 2005; Howarth 2008; Wurtsbaugh et al. 2019). Excess nitrogen reduces biodiversity by favoring nutrient-tolerant species over sensitive ones and alters food webs (Meunier et al. 2016), affecting drinking water (Ghaly and Ramakrishnan 2015; Westerhoff and Mash 2002), fisheries (Prakash and Khanam 2021), and recreation services (Ghaly and Ramakrishnan 2015).

Identifying and implementing effective measures to reduce local and regional nitrogen pollution amid population growth, development, and water scarcity remains a matter of debate (Qadir et al. 2007; Vorosmarty et al. 2010). One such internationally applied measure to minimize water pollution has been identified as water quality offsetting (Liu and Brouwer 2023; Shortle 2013) (see Box 1). Water quality offsetting can be used when a pollution impact is compensated by reducing pollution elsewhere, achieving no net decline in water quality compared to what would have occurred without the impact, while still allowing economic development (Hundloe 2021; Miller et al. 2015) (see Box 2). Nitrogen offsetting has been proposed as an important strategy to prevent the degradation of aquatic ecosystems, including coral reefs, which are among the most threatened globally (Beyer et al. 2018; Hoegh-Guldberg et al. 2007) despite supporting some of the highest levels of marine biodiversity (Reaka-Kudla 1997). In Australia, efforts are underway to develop and implement nitrogen offset programs, particularly in the Great Barrier Reef Catchment Area (GBRCA), where excess nutrient runoff from agriculture and other land uses poses a major threat to water quality (GBRMPA 2019). Using the GBRCA as an example, we develop a framework to guide nitrogen offset strategies in tropical and subtropical regions worldwide.

Box 1 Context of Nitrogen Offsetting: Background and Challenges Environmental offsetting has been widely implemented within Australia (Gibbons et al. 2018; Hayes and Morrison-Saunders 2007; Miller et al. 2015) and internationally (Droste 2022; Lovell 2010; Shortle 2013; Theis 2020). The concept has been mainly applied in the context of carbon emissions to mitigate global warming (Hyams and Fawcett 2013; Lovell 2010) and biodiversity management to compensate for impacts on species or ecosystems (Bull et al. 2013; Gibbons et al. 2018; Maron et al. 2015; Ten Kate et al. 2004). However, the widespread adoption of water quality offsetting has been challenging due to difficulties in identifying the most appropriate offset types for different pollutants across catchments, uncertainties regarding their effectiveness, as well as economic, social, and political barriers to their implementation (Fisher-Vanden and Olmstead 2013; Liu and Brouwer 2023; Lu et al. 2023).

Defining what constitutes a suitable water quality offset is fundamental to effective offsetting. In principle, any intervention that demonstrably reduces nitrogen loads to aquatic ecosystems can be considered a nitrogen offset (State of Queensland 2019). These include both direct water treatment methods and measures that reduce nitrogen inputs at the source, such as optimizing fertilizer use in agriculture (Fang et al. 2005) or controlling riverbank erosion (State of Queensland 2019). However, there is limited consensus on suitable offset types across existing water quality offsetting programs (State of Queensland 2019). This reflects differences in policies and regulations, including variation in targeted pollutants (e.g., TN, DIN, or other nitrogen forms), as well as socio-economic and governance contexts that influence the feasibility of offset options, and geographic differences in climatic and ecological conditions that affect offset equivalence (Corrales et al. 2013; Fisher-Vanden and Olmstead 2013; Horan and Shortle 2011). Similar uncertainty exists regarding which pollution sources are appropriate for offsetting. Although current policies in the GBRCA focus primarily on point sources (State of Queensland 2019), nitrogen reductions could, in principle, be applied to both point and non-point sources (Fang et al. 2005; Stephenson and Shabman 2017; Zhou et al. 2016).

Box 2 Equivalence in Offsetting Environmental offsets rely on the principle of environmental equivalence (Bull et al. 2016; Hundloe 2021; Maron et al. 2012; Miller et al. 2015). Environmental equivalence in offsetting can be defined as an equal transaction in offset exchange to achieve a no net loss in terms of type, amount, and duration of an impact (i.e., like-for-like exchange); meaning that what is impacted should be benefited elsewhere, that gain should be at least as large as the loss, and the benefits should have the same the duration of the impact (Miller et al. 2015). Finding equivalence in nutrient offsetting is not straightforward due to uncertainties in predicting environmental outcomes, which arise from the choice of offset metric, the performance of the offset method, and the variability of natural environments (Clarke and Bradford 2014; Moilanen et al. 2009). Nitrogen offsetting is particularly challenging because the processes that reduce a specific form of nitrogen differ across locations, influenced by both natural and anthropogenic conditions within and across catchments (Lu et al. 2023).

Equivalence ratios have been incorporated into legislation to facilitate the implementation of suitable offsets by providing a standard method for calculating the required offset size (Clarke and Bradford 2014). In the context of the GBRCA and the associated Reef Protection Regulations and Point Source Water Quality Offsets Policy (State of Queensland 2019), the total nitrogen (TN) annual load has been defined as the offset metric, and two types of equivalence ratios are specified: delivery ratios and environmental equivalence ratios. Delivery ratios, which account for uncertainty in achieving an equivalent pollution reduction, are set at 1:1.5, meaning that the amount required to be offset is 50% greater than the pollutant load at the impacted site. Environmental equivalence ratios, which account for the like-for-like chemical exchange between the pollutant release point and the water quality offset, are set at 1:1 (State of Queensland 2019). While nitrogen offsetting could target each form of nitrogen individually, determining the appropriate equivalence ratios requires an understanding of the hydrological and biological processes that control the consumption and transformation of different nitrogen forms within and across catchments (Lu et al. 2023).

Developing equivalence ratios that accurately reflect environmental equivalence for different forms of nitrogen across lacustrine and palustrine ecosystems in the GBRCA is essential to ensure that nitrogen reductions benefit the same ecosystems affected by pollution and that the magnitude of benefits matches the impacts (Lu et al. 2023). A further critical gap in current research is the limited consideration of climate change impacts on offset effectiveness. Natural variability, including extreme rainfall events, already affects nitrogen removal performance, and projected changes in precipitation patterns and hydrological regimes are likely to increase these uncertainties (Feng et al. 2013; Giorgi et al. 2019). Incorporating environmental modeling that accounts for climate change projections into nitrogen offset planning is therefore necessary to ensure offsets remain effective under future conditions, particularly in regions prone to increased rainfall variability and extreme weather events.

### Nitrogen Pollution in the GBRCA

The Great Barrier Reef World Heritage Area (GBRWHA) spans approximately 346,000 km² and protects the largest and one of the most biodiverse coral reef systems globally (GBRMPA 2019). The GBRWHA supports multiple marine ecosystems, including seagrass beds, *Halimeda* banks, and diverse pelagic and coastal habitats. These ecosystems support multiple ecosystem services and contribute significantly to the Australian economy, particularly through fisheries and tourism (Stoeckl et al. 2011), with an estimated total value of AUD 56 billion and an annual economic contribution of AUD 6.4 billion (O’Mahoney et al. 2017). However, the GBR faces multiple anthropogenic pressures, many originating from the Great Barrier Reef Catchment Area (GBRCA). Covering approximately 424,000 km² across six natural resource management regions, the GBRCA has a population of about one million people and has experienced extensive land clearing and ecosystem degradation (GBRMPA 2019). These activities have increased pollutant loads—including sediments, nitrogen, phosphorus, and pesticides—reaching the GBR lagoon from 35 river basins (Kroon et al. 2016; Waterhouse et al. 2012). In the GBRCA, nitrogen pollution originates from both non-point sources (e.g., agricultural runoff, erosion, urban stormwater) and point sources (e.g., mining, meat processing, aquaculture, wastewater treatment plants) (GBRMPA 2019). Nitrogen has been shown to affect coral reef survival in the region, as its excess increases the vulnerability of coral reefs to bleaching, promotes macroalgae and phytoplankton growth, fosters pathogen outbreaks (Zhao et al. 2021), and contributes to the proliferation of coral predators (Brodie et al. 2005).

In 2019, the Queensland Government introduced the Reef Protection Regulations and the Point Source Water Quality Offsets Policy to avoid, minimize, and, when necessary, offset nitrogen and sediment discharge into the GBRWHA (State of Queensland 2019). Based on the Policy, offset types (i.e., actions to reduce nitrogen load baselines in different locations relative to nitrogen pollutant sources) can consist of point or non-point source offsets. These include, but are not limited to, riparian area restoration, streambank and gully restoration, constructed or remediated wetlands, bioremediation technologies including bioreactors, riparian fencing for stock exclusion, reduction of on-farm nutrient runoff through improved fertilizer application management above minimum standards, improved grazing land management practices above minimum standards, and water sensitive urban designs beyond the stormwater management design objectives under the State Planning Policy 2017 (State of Queensland 2019). The Queensland government has also proposed a no-net-increase target for nitrogen loads reaching the GBRWHA (Commonwealth of Australia 2021; McMahon et al. 2023), with discussions underway regarding a market-based water quality offset system (Australian Government 2022; Burford and Lu 2023).

### A Framework for Nitrogen Offsetting in Tropical and Subtropical Regions

Using the Great Barrier Reef Catchment Area (GBRCA) as an example of a region where water quality offsetting is ongoing based on nitrogen, we developed a framework to guide the identification, selection, and implementation of effective nitrogen offset strategies in tropical and subtropical regions worldwide. The framework is grounded in the premise that effective nitrogen offset design requires integrating three key elements: (i) scientific evidence on the performance of nitrogen mitigation strategies (Haddaway et al. 2018; Newell et al. 2011; Randall et al. 2015), (ii) clear criteria for selecting appropriate offset types based on environmental, social, and technical considerations (Horan and Shortle 2011; Shortle 2013; Zapata et al. 2023), and (iii) adaptive management processes to address uncertainty and variability in environmental outcomes (Spicer et al. 2021). Accordingly, the framework comprises three interrelated components: (1) assessment of water quality improvement methods for nitrogen reduction in the GBRCA and other tropical and subtropical regions based on performance (i.e., efficacy, effectiveness, and efficiency); (2) selection of suitable offset types based on their performance, co-benefits, and spatial and technical feasibility; and (3) integration of adaptive management strategies, Geographic Information Systems (GIS) tools, and monitoring systems to strengthen the effectiveness of nitrogen offsetting. Together, these components provide a structured, evidence-based approach for designing nitrogen offset strategies applicable across diverse aquatic ecosystems and pollutants worldwide.

## Methods

### Literature Review on Water Quality Improvement Methods

As the first step of our proposed framework, we conducted a comprehensive literature review of scientific databases to identify methods for reducing nitrogen inputs to aquatic ecosystems, using a range of search terms (i.e., remove, abate, reduce, and limit) to capture relevant literature. Performance of offset types was assessed based on three metrics: efficacy, effectiveness, and efficiency. Efficacy was defined as the ability of water quality improvement methods to reduce nitrogen concentrations or loads under controlled conditions, including laboratory experiments. Effectiveness was defined as their ability to reduce nitrogen concentrations or loads under real-world conditions and natural weather variability. Efficiency was defined as nitrogen reduction achieved per dollar. Water quality improvement methods correspond to the offsets applied to counteract pollution impacts, including point source offsets (e.g., wastewater treatment plants) and non-point source offsets (e.g., optimized fertilizer use in agricultural lands).

To examine relevant manuscripts within the GBRCA, we used the following combinations of keywords in the scientific database Web of Science: TOPIC = (water AND quality) AND (remov* OR abat* OR reduc* OR limit*) AND (nitrogen OR DIN) AND (efficac* OR effective* OR efficien*) AND (“Great Barrier Reef”). This led to a total of 34 publications, of which 12 publications were selected as suitable for further assessment as they explored the performance of water quality improvement methods for nitrogen in the GBRCA. Then, we used the following combinations of keywords to examine manuscripts developed in tropical and subtropical areas globally: TOPIC = (water AND quality) AND (remov* OR abat* OR reduc* OR limit*) AND (nitrogen OR DIN) AND (efficac* OR effective* OR efficien*) AND (tropic* OR subtropic*). This search yielded 298 publications, of which 59 were selected for further analysis based on their direct relevance to the performance of water quality improvement methods for nitrogen in tropical or subtropical regions. Studies were included regardless of the presence of experimental controls, as real-world evaluations often lack them. In total, 66 studies were deemed suitable for further assessment (Table S1 in Supplementary Information).

We categorized studies based on their performance metric (efficacy, effectiveness, or efficiency). In cases where total DIN was not reported, but individual constituents were available (ammonium, ammonia, nitrite, nitrate) (see Box 3), we used the most abundant form as a conservative proxy for total DIN to retain as many data points as possible in the analysis. When not directly available, the nitrogen reduction percentage was calculated by comparing the inflow and outflow concentrations or loads, using the formula:

$${Reduction}\,{Percentage}=(({Inflow}-{Outflow})/{Inflow})\times 100$$

where ${Inflow}$ is the concentration or load of nitrogen entering the system and ${Outflow}$ is the concentration or load of nitrogen leaving the system. Whenever possible, data points included average, minimum, and maximum values to better represent nitrogen reduction dynamics, capturing variability in inflow and outflow conditions and providing a more accurate assessment of nitrogen reduction across different scenarios.

To visualize the variation in TN and DIN reduction across different water quality improvement methods, we used boxplots. We compared nitrogen reduction across treatment methods for efficacy and effectiveness separately, and then combined them for a broader comparison. Next, we assessed nitrogen removal while accounting for sample size differences by calculating Hedges’ *g* standardized mean difference (i.e., effect size analysis) (Durlak 2009) between inflow and outflow nitrogen values (measured as concentration or load). Hedges’ *g* provides a standardized and bias-corrected measure of effect size, allowing robust comparison and synthesis across studies. Hedges’ *g* was calculated using the following formula:

$$g=\frac{{M}_{1}-{M}_{2}}{{{SD}}_{{pooled}}}\times \left(\frac{N-3}{N-2.25}\right)\times \sqrt{\frac{N-2}{N\,}}$$

where ${M}_{1}$ is the mean inflow nitrogen value, ${M}_{2}$ is the mean outflow nitrogen value, ${{SD}}_{{pooled}}$ is the pooled standard deviation (see below), and $N$ is the total number of inflow and outflow samples. The pooled standard deviation was calculated as:

$${{SD}}_{{pooled}}=\sqrt{\frac{{{SD}}_{1}^{2}+{{SD}}_{2}^{2}}{2\,}}$$

where ${{SD}}_{1}$ is the standard deviation of inflow nitrogen values and ${{SD}}_{2}$ is the standard deviation of outflow nitrogen values. A Hedges’ *g* of 1 indicates that the groups differ by one standard deviation, while a value of 2 indicates a difference of two standard deviations. Effect sizes are typically categorized as small (0.2), medium (0.5), and large (greater than 0.8) (Bakker et al. 2019). Higher effect sizes indicate greater nitrogen removal efficiency by the water quality treatment method.

For the efficiency metric, we could only visualize variation in total nitrogen (TN) and dissolved inorganic nitrogen (DIN) reduction using studies from Australia, due to limited comparable studies from other regions.

Box 3 Natural and Anthropogenic Forms of Nitrogen Nitrogen gas (N_2_) is naturally transformed into reactive forms of nitrogen through biological fixation mediated by microorganisms (Fowler et al. 2013). However, human activities, such as burning fossil fuels, fertilizer production, and agriculture, have significantly increased the release of reactive nitrogen (Canfield et al. 2010; Galloway et al. 2008; UNESCO 2021), affecting ecosystems, biodiversity, and air quality. Excess nitrogen contributes to eutrophication (Hautier et al. 2009) and the formation of tropospheric ozone, both of which negatively affect ecosystems and human health (Lefohn et al. 2018). Additionally, nitrous oxide (N₂O) produced from reactive nitrogen has a global warming potential approximately 300 times greater than an equivalent mass of carbon dioxide (Crutzen et al. 2008).

In aquatic ecosystems, N_2_ is converted into reactive nitrogen through biological fixation, and additional nitrogen from human activities enters these systems via atmospheric deposition, land-based runoff, leaching, and direct point source discharges (Xia et al. 2018). Reactive nitrogen exists in various forms, including dissolved inorganic nitrogen (DIN), which comprises ammonium (NH_4_^+^), ammonia (NH_3_), nitrate (NO_3_^−^), and nitrite (NO_2_^−^); dissolved organic nitrogen (DON), which consists of nitrogen-containing organic compounds; and particulate organic and inorganic nitrogen (Meybeck 1982; Seitzinger et al. 2010; Sipler and Bronk 2015). Although DIN is generally assumed to be the primary form influencing phytoplankton growth and is commonly used as an indicator of eutrophication, research indicates that DON can also significantly affect phytoplankton productivity (Bronk et al. 2007; Mackay et al. 2025).

The transformation of nitrogen includes the degradation of organic nitrogen into NH_4_^+^ (i.e., ammonification), the oxidation of NH_4_^+^ into NO_3_^−^ (i.e., nitrification), the reduction of NO_3_^−^ into N_2_ (i.e., denitrification), and the reduction of NO_2_^−^ into N_2_ in the presence of NH_4_^+^ (i.e., anaerobic ammonium oxidation, or anammox) (Xia et al. 2018). These processes vary across catchments due to different microorganism communities, hydrological regimes, water temperature, levels of land-use change, and pollution (Gergel et al. 2005; Meybeck 1982; Xia et al. 2018). Effective planning to limit nitrogen pollution is therefore context-specific and requires an understanding of the ecological interactions that drive nitrogen transformations within and across catchments (Lu et al. 2023).

### Integrating Co-Benefits, Feasibility, and Adaptive Management

We examined co-benefits, spatial and technical feasibility using relevant literature and case studies, while also exploring the integration of adaptive management approaches, geographic information systems, and robust monitoring mechanisms to enhance the success of nitrogen offset strategies, ensure accountability, and support long-term environmental sustainability in nitrogen offset implementation.

## Reviewing Water Quality Improvement Strategies: Performance Metrics

**Fig. 1:**
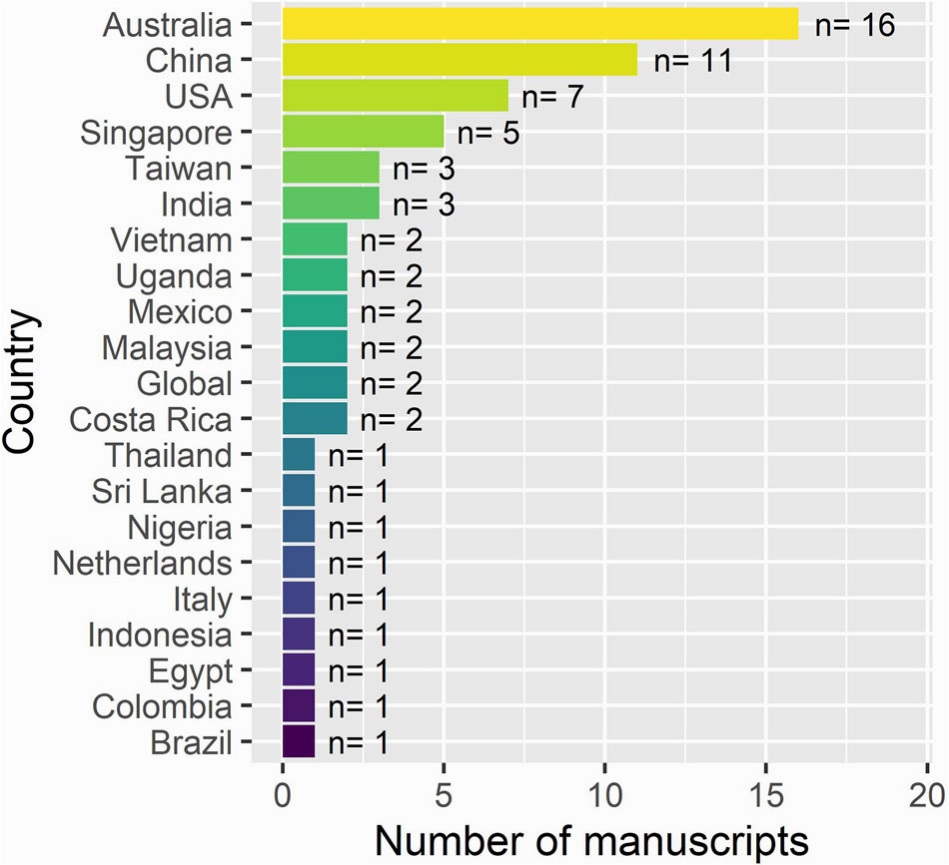
Locations (by country) of the 66 studies that analyzed water quality improvement methods in tropical and subtropical areas globally. A study from the Netherlands was included as it simulated tropical conditions

**Fig. 2:**
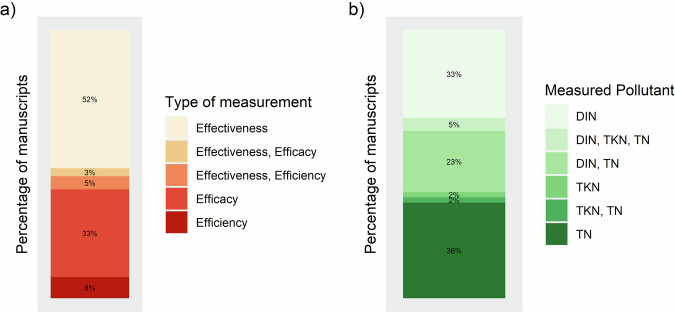
**a** Performance metrics and **b** the main forms of nitrogen included in the 66 studies analyzing water quality improvement methods in tropical and subtropical areas globally

Assessed studies were conducted in 20 countries across Africa, Asia, the Americas, Oceania, and subtropical Europe (Fig. 1; Table S1 in Supplementary Information), with Australia (24%), China (17%), and the USA (11%) being the most represented. One study simulating tropical conditions was included despite occurring in the Netherlands. Most studies evaluated water quality improvement based on effectiveness (52%), followed by efficacy (33%), while only a few considered efficiency (8%) or multiple performance metrics (8%) (Fig. 2a). Studies most commonly analyzed total nitrogen (TN) alone (36%), followed by dissolved inorganic nitrogen (DIN) alone (33%), with fewer examining both TN and DIN (23%). Total Kjeldahl nitrogen (TKN; which equals total organic nitrogen, ammonia, and ammonium, excluding nitrite and nitrate) or other nitrogen forms were considered in 9% of studies (Fig. 2b).

Treatment ponds/wetlands were the most studied method (53% of references), encompassing wastewater, agriculture, aquaculture, urban stormwater, and unspecified ponds/wetlands. Improved agricultural management practices ranked second (18%), including fertilizer management, best management practices (BMPs), agroforestry, and combined approaches. Conservation of native ecosystems (11%) focused on wetland preservation, while other bioremediation systems (8%) involved bioreactors. Fewer studies examined improved sewage treatment plants, land-use conversion, terrestrial habitat restoration based on riparian buffers, or non-biological systems such as adsorbents. Through this assessment, we identified water quality improvement methods that could supplement existing nitrogen offset types outlined in the 2019 Point Source Water Quality Offsets Policy for the GBRCA (Australian Government 2018; State of Queensland 2019), the Aquatic Ecosystem Rehabilitation Process, and the Queensland River Rehabilitation Management Guideline (Burton et al. 2022), as well as similar international policies (Table 1).

**Table 1:** List of water quality improvement methods potentially suitable as nitrogen offsets, identified from 66 studies analyzing water quality improvement in tropical and subtropical regions globally

General offset type	Specific offset type	Detailed offset type	Point Source Water Quality Offsets Policy (2019)	Aquatic ecosystem rehabilitation process and the Queensland River Rehabilitation Management guideline
Conservation of native ecosystems	Conservation of native ecosystems based on existing wetlands	Conservation of native ecosystems based on existing wetlands – Estuaries		
		Conservation of native ecosystems based on existing wetlands – Lakes/reservoirs		
		Conservation of native ecosystems based on existing wetlands – Riverine wetlands		
Construction of non-biological remediation systems	Construction of non-biological remediation systems based on adsorbents	Construction of non-biological remediation systems based on adsorbents – Various	Bioremediation technologies (point source)	
Construction of other bioremediation systems	Construction of other bioremediation systems based on bioreactors	Construction of bioreactor systems – Denitrifying bioreactors		
		Construction of bioreactor systems – Microalgal photobioreactors		
		Construction of bioreactor systems – Others		
Construction of sewage treatment plants	Improved sewage treatment plants	Improved sewage treatment plants – Downflow-upflow biological aerated filter		
		Improved sewage treatment plants – Others		
		Improved sewage treatment plants – T1 to T3		
Construction of treatment ponds/wetlands	Construction of agricultural treatment ponds/wetlands	Construction of agricultural treatment ponds/wetlands – Drains/ditches/strips	Constructed or remediated wetlands	Wetland restoration/rehabilitation
		Construction of agricultural treatment ponds/wetlands – Others		
	Construction of aquaculture treatment ponds/wetlands	Construction of aquaculture treatment ponds/wetlands – Fish breeding		
		Construction of aquaculture treatment ponds/wetlands – Shrimp breeding		
	Construction of non-specified treatment ponds/wetlands	Construction of non-specified treatment ponds/wetlands – Various		
	Construction of urban stormwater treatment ponds/wetlands	Construction of urban stormwater treatment ponds/wetlands – Modular bioretention tree	Water-sensitive urban designs above the stormwater management design objectives	
		Construction of urban stormwater treatment ponds/wetlands – Various		
	Construction of wastewater treatment ponds/wetlands	Construction of wastewater treatment ponds/wetlands – Drains/ditches	Constructed or remediated wetlands	
		Construction of wastewater treatment ponds/wetlands – Submerged macrophytes		
		Construction of wastewater treatment ponds/wetlands – Subsurface flow/floating macrophytes/surface flow/irrigated forest		
Improved agricultural management practices	Improved agricultural management practices (Best Management Practices)	Improved agricultural management practices (Best Management Practices) – ABCD	Reduction of on-farm nutrient runoff through improved fertilizer application above minimum standards	
		Improved agricultural management practices (Best Management Practices) – Various		
	Improved agricultural management practices (Others)	Improved agricultural management practices (Others) – Nutrient/fallow/tillage		
	Improved agricultural management practices based on agroforestry	Improved agricultural management practices based on agroforestry – Various		
	Improved agricultural management practices based on fertilizers	Improved agricultural management practices based on fertilizers – Various		
Land-use conversion	Land-use conversion to less intensive production systems	Land-use conversion to less intensive production systems – Sugarcane to grazing		
	Land-use conversion to native ecosystems	Land-use conversion to native ecosystems – Sugarcane to native (Various)		
		Land-use conversion to native ecosystems – Sugarcane to wetlands		
Terrestrial habitat restoration/rehabilitation	Terrestrial habitat restoration/rehabilitation based on the creation of riparian buffers	Terrestrial habitat restoration/rehabilitation based on the creation of riparian buffers – Strips	Riparian area restoration	

Equivalent terms are based on the Point Source Water Quality Offsets Policy in 2019 (State of Queensland 2019), the Aquatic Ecosystem Rehabilitation Process, and the Queensland River Rehabilitation Management guideline (Burton et al. 2022). Improved sewage treatment plants (T1–T3) refer to systems with primary, secondary, and tertiary treatment stages only, excluding quaternary treatment targeting micropollutants (e.g., pharmaceuticals and pesticides). Non-specified treatment wetlands include systems not classified or inferred as agricultural, aquacultural, urban stormwater, or wastewater treatment wetlands. Best management practices (BMPs) encompass a range of government-defined recommendations to reduce pollution from rural settings. Improved agricultural management practices (BMPs–ABCD) refer to a framework developed in Australia that classifies practices from degrading (D), to common (C), best (B), and aspirational (A), based on their impact on water quality, expected resource condition over time, community acceptance, and feasibility of adoption.

### Nitrogen Removal by Percentage (Efficacy and Effectiveness)

**Fig. 3:**
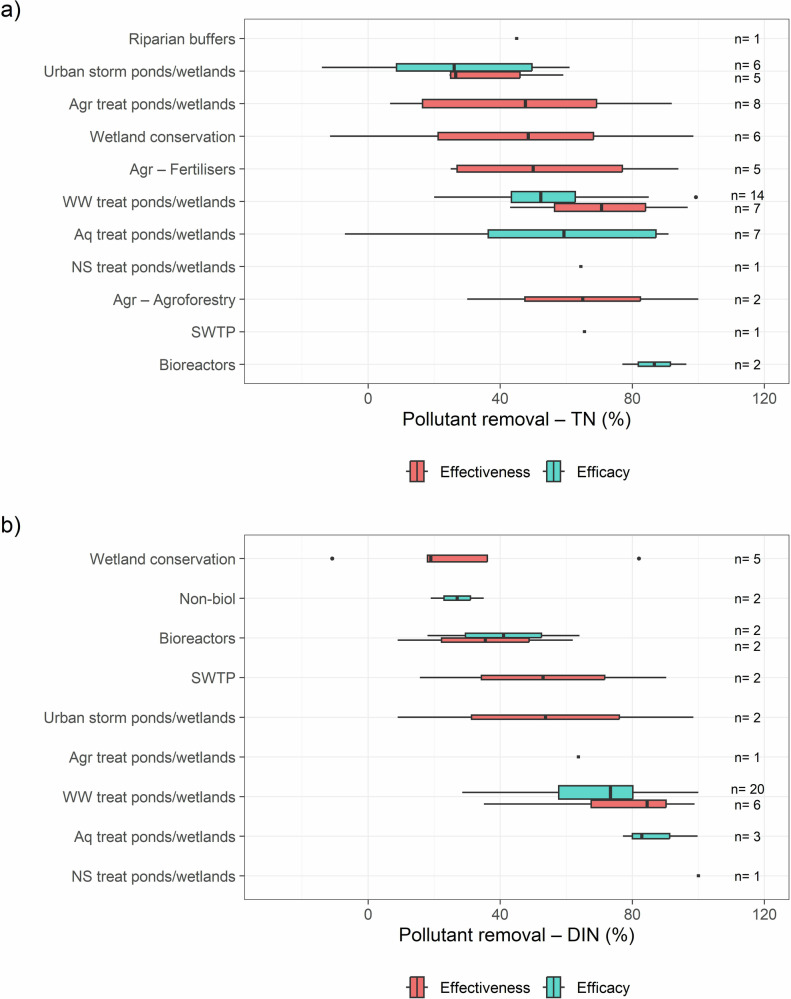
Efficacy/effectiveness, as a percentage of reduction of **a** Total Nitrogen (TN) and **b** Dissolved Inorganic Nitrogen (DIN) per offset type across studies analyzing quality improvement methods in tropical and subtropical areas globally. The number of measurements (n) is shown on the right of each boxplot. Agr agricultural, Aq aquacultural, BMPs best management practices, Non-biol non-biological, NS Non-specified, storm stormwater, SWTP sewage treatment plants, treat treatment, WW wastewater

Due to limited data on other water quality improvement methods, we could only compare the efficacy and effectiveness of total nitrogen (TN) removal within urban stormwater and wastewater treatment ponds/wetlands (Fig. 3a), and of dissolved inorganic nitrogen (DIN) removal within bioreactors and wastewater treatment ponds/wetlands (Fig. 3b). Although higher nitrogen removal is generally expected under controlled conditions, our analysis did not reveal a clear trend. Only two studies, both on urban stormwater ponds/wetlands, directly compared efficacy and effectiveness at the same site, finding increased nitrogen removal during experimental tests (Lim et al. 2021; Neo et al. 2022) and thus suggesting controlled conditions may overestimate real-world performance. The overall lack of trend likely reflects environmental variability across studies and the limited evidence base.

**Fig. 4:**
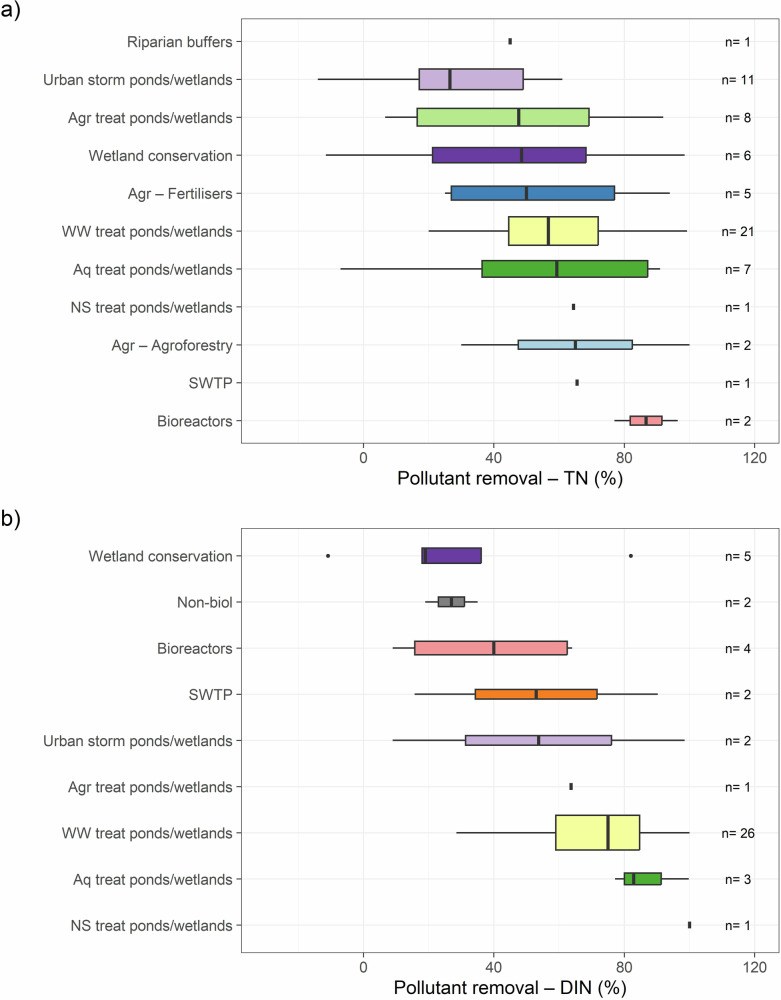
Merged efficacy/effectiveness per offset type as a percentage of reduction of **a** Total Nitrogen (TN) and **b** Dissolved Inorganic Nitrogen (DIN) across studies analyzing quality improvement methods in tropical and subtropical areas globally. The number of measurements (n) is shown on the right of each boxplot. Agr agricultural, Aq aquacultural, BMPs best management practices, Non-biol non-biological, NS Non-specified, storm stormwater, SWTP sewage treatment plants, treat treatment, WW wastewater

When combining efficacy and effectiveness across methods, bioreactors showed the highest TN reduction (Fig. 4a). However, as these studies were conducted under controlled conditions, their performance may be overestimated, highlighting the need for field-based trials to assess their effectiveness under variable environments. In contrast, treatment ponds/wetlands consistently delivered the highest DIN reduction (Fig. 4b), likely driven by microbial, algal, and plant uptake processes (Jordan et al. 2011; Lee et al. 2009). These results emphasize the importance of matching offset strategies to specific nitrogen components and expanding comparative studies that evaluate both efficacy and effectiveness within the same sites.

### Nitrogen Removal by Effect Size (Efficacy and Effectiveness)

**Fig. 5:**
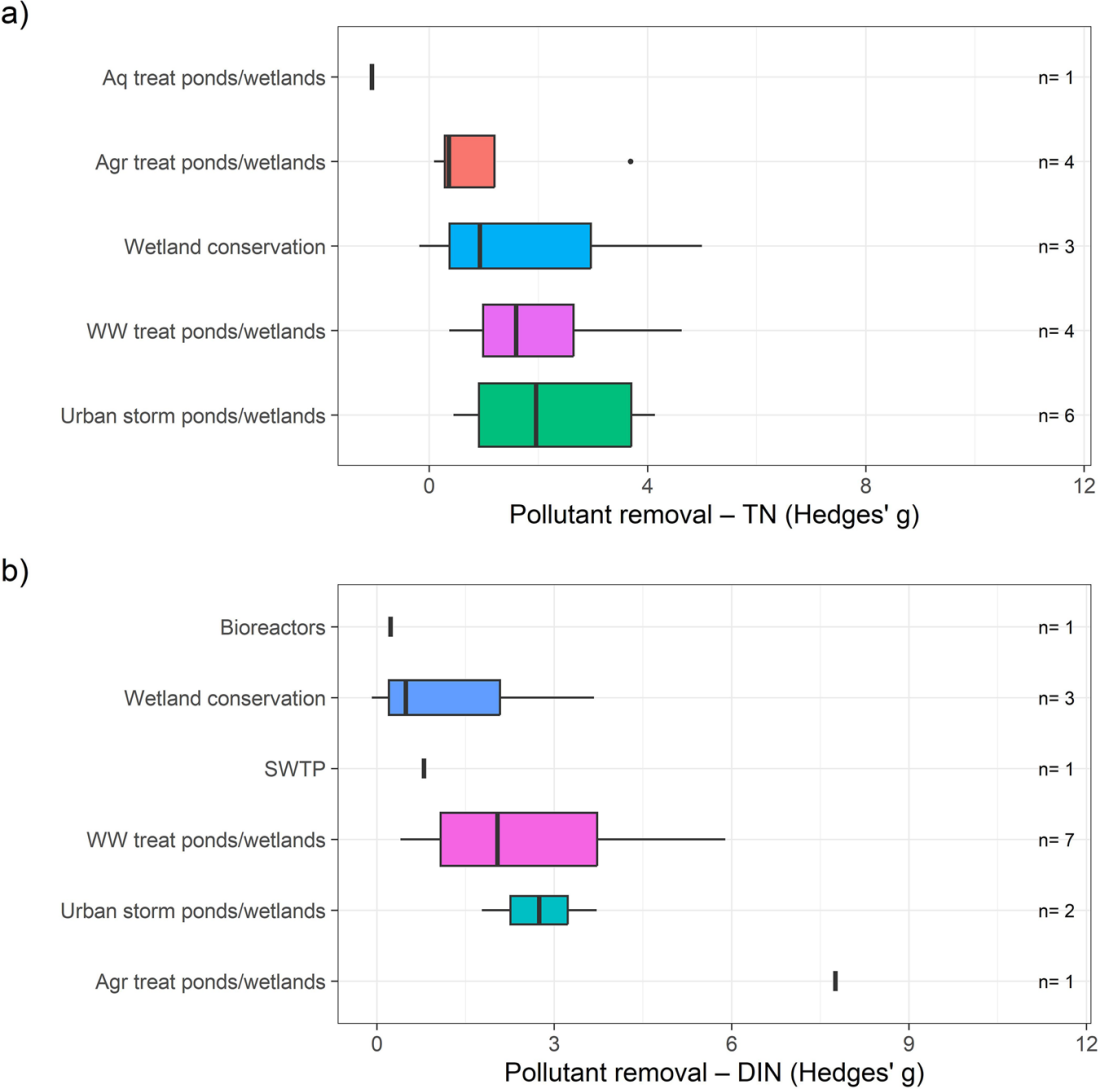
Effect sizes per offset type for **a** Total Nitrogen (TN) and **b** Dissolved Inorganic Nitrogen (DIN) across 25 studies analyzing water quality improvement methods in tropical and subtropical areas globally. The number of measurements (n) is shown on the right of each boxplot. Agr agricultural, Aq aquacultural, storm stormwater, SWTP sewage treatment plants, treat treatment, WW wastewater

Effect size calculations were possible for a subset of 25 studies, including bioreactors, conservation of wetlands, treatment ponds/wetlands (covering agriculture, aquaculture, urban stormwater, and wastewater treatment ponds/wetlands), and improved sewage treatment plants. For TN removal, the highest Hedges’ $g$ values, indicating greater nitrogen removal, were observed in urban stormwater treatment ponds/wetlands (Fig. 5a). For DIN removal, agricultural treatment ponds/wetlands had the highest Hedges’ $g$ values (Fig. 5b). While effect size analysis standardizes performance across studies with varying sample sizes (Bakker et al. 2019), the lack of consistent reporting of raw inflow and outflow nitrogen values limits the reliability of cross-study comparisons. Therefore, our synthesis focuses on identifying consistent patterns in relative performance rather than precise quantitative comparisons. We recommend that future studies report raw inflow and outflow nitrogen values (concentration or load) to enable robust effect size calculations and meta-analyses, strengthening the evidence base for nitrogen offset frameworks.

### Nitrogen Removal by Efficiency

**Fig. 6:**
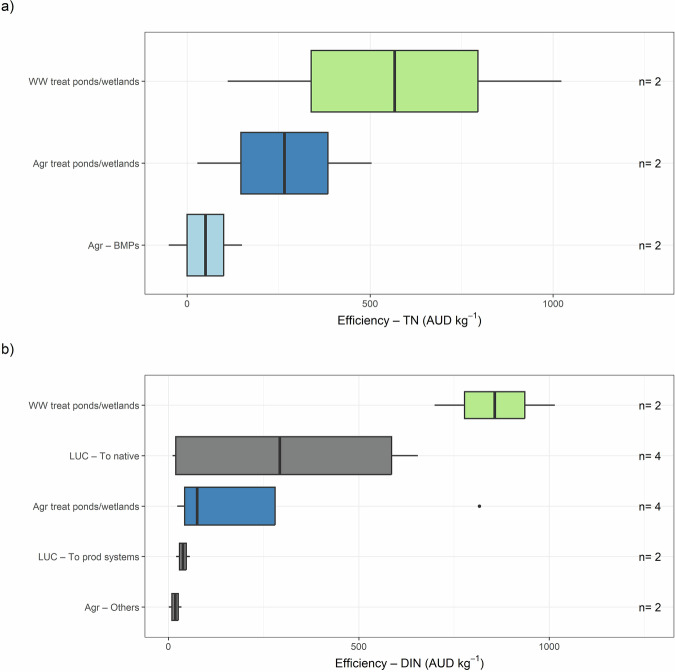
Efficiency per offset type for **a** Total Nitrogen (TN) and **b** Dissolved Inorganic Nitrogen (DIN) across three studies developed in Australia. The number of measurements (n) is shown on the right of each boxplot. Agr agricultural, BMPs best management practices, LUC land-use change, prod production, treat treatment, WW wastewater

Except for one study from the USA, all efficiency evaluations were conducted in Australia, reflecting the limited availability of comparable studies elsewhere. Among these studies, agricultural BMPs were the most cost-effective for TN reduction compared to agricultural and wastewater treatment ponds/wetlands (Fig. 6a). For DIN reduction, the most cost-effective approaches were combined systems incorporating nutrient, fallow, and tillage management, as well as land-use conversion to less intensive production systems (Fig. 6b). These methods outperformed land-use changes to native ecosystems or agricultural and wastewater treatment ponds/wetlands. However, they require large land areas, limiting their applicability in small or highly polluted basins (Stephenson et al. 2010).

### Identifying the Best Offset Type Based on its Performance

Identifying the most suitable water quality improvement methods for nitrogen reduction in tropical and subtropical conditions, as well as in the GBRCA, remains challenging. Most studies are concentrated in Australia, China, and the USA, while countries in Africa, Central and South America, Southeast Asia, and Oceania remain underrepresented despite their high environmental variability. This geographic bias limits the assessment of nitrogen reduction methods across diverse tropical and subtropical environments. Comparability is further constrained by inconsistencies in evaluation approaches (effectiveness, efficacy, or efficiency) and nitrogen forms measured (TN, DIN, their constituents, or total Kjeldahl nitrogen), reducing the availability of comparable studies for meaningful cross-analysis. Additionally, the lack of raw data in most studies prevents the application of robust statistical methods such as the Hedges’ $g$ effect size. Research is also heavily skewed toward treatment ponds/wetlands and agricultural management practices, with other strategies receiving limited attention. For example, only a few studies examine improved sewage treatment plants (*n* = 3), land-use conversion to less intensive production systems (*n* = 2), habitat restoration based on riparian buffers (*n* = 1), or non-biological systems such as adsorbents (*n* = 1). These gaps highlight the need for broader geographic representation, standardized evaluation methods, and increased exploration of diverse nitrogen reduction strategies, particularly those providing additional environmental benefits (see section on co-benefits).

Despite biases in data, our analysis indicates that while bioreactors achieve strong TN reduction, treatment ponds/wetlands are more multifunctional and widely applicable. In addition to DIN removal, they provide co-benefits such as habitat provision, carbon sequestration, overall water quality improvement, and flood regulation (An and Verhoeven 2019; Spieles 2022). However, the most efficacious or effective methods are not necessarily the most efficient. Evidence from Australian studies suggests that agricultural practices are the most cost-effective for reducing TN and DIN, while land-use conversion to less intensive production systems is particularly cost-effective for DIN reduction. The recognition of wetlands as a nature-based solution in tropical and subtropical regions (Ferreira et al. 2023), including the GBRCA, further supports their role in integrated water quality management alongside cost-effective agricultural approaches.

As no single method is likely to achieve nitrogen reduction targets alone, we advocate combining offset strategies based on both effectiveness and efficiency at the basin scale (Liu et al. 2022). This approach helps avoid directly extrapolating results from controlled conditions to real-world settings, while also ensuring that efficiency is evaluated within the socio-economic context of each region. Trade-offs between water quality improvement methods and land use must also be carefully considered, particularly for offsets requiring extensive land areas (e.g., agricultural management practices) (Stephenson et al. 2010). Finally, further research is needed on underrepresented nitrogen reduction approaches, including habitat restoration based on riparian buffers, which can deliver co-benefits as habitat provision and carbon storage (Macreadie et al. 2021; Strassburg et al. 2020).

## Identifying Suitable Offset Strategies: Performance, Co-Benefits, And Spatial And Technical Feasibility

Identifying suitable nitrogen offset strategies requires evaluating potential mitigation actions across multiple dimensions, including their performance, associated environmental and social co-benefits, and technical and spatial feasibility.

### Performance

Our literature review indicates that the ability of water quality improvement methods to reduce nitrogen is a primary criterion for selecting offset types. We propose that this selection should be based on their effectiveness (performance under real-world conditions) and efficiency (i.e., considering cost-effectiveness) under local environmental conditions. Efficacy data from controlled experiments can be used cautiously or combined with environmental modeling to estimate potential nitrogen reductions when effectiveness or efficiency data are lacking.

To assess the effectiveness of water quality offsets, repeated measures would be particularly useful to capture the temporal variability in nitrogen reduction (i.e., long-term monitoring), as a consequence, for instance, of changes in hydrological conditions and climatic fluctuations that have been shown to affect nitrogen reduction (Newham et al. 2024; Puzyreva et al. 2019). By compiling long-term monitoring from previously implemented offsets (for instance, from comprehensive water quality offset registers or other water quality improvement projects), uncertainties in nitrogen removal effectiveness can be reduced, leading to more reliable offset planning and better predictive modeling.

### Co-benefits

Along with performance, offset strategies can be prioritized for their environmental and social co-benefits, as they can contribute to reducing the interacting pressures that drive ecological degradation and align with the local and regional policies on environmental sustainability, such as the Sustainable Development Goals (UN 2018). Environmental co-benefits include the provision of habitat for native terrestrial and aquatic species, carbon sequestration, flooding resilience, reduction in other pollutant sources including pesticides and nutrients besides nitrogen, and increased air quality (Adame et al. 2019; Hagger et al. 2022; Miettinen et al. 2012; Smith et al. 2013). For example, habitat restoration can improve water quality while supporting biodiversity and carbon storage (Macreadie et al. 2021; Strassburg et al. 2020). In the context of the GBRCA, Hagger et al. (2022) reported that within the Wet Tropics region, approximately 5000 ha of agricultural land—including sugarcane fields, grazing areas, and abandoned aquaculture sites—could be restored to coastal wetlands, such as *Melaleuca* wetlands, vine forests, mangroves, sedgelands, freshwater waterholes, and estuaries. This restoration has the potential to sequester around 221,000 tonnes of carbon dioxide-equivalent (CO₂-e) annually, while simultaneously improving water quality, enhancing biodiversity, and increasing the value of coastal fisheries.

Nitrogen offsetting can also provide social co-benefits. For example, riverine restoration can improve air and water quality for surrounding communities, with positive implications for human health (Basak et al. 2021; Houlton et al. 2019; Kaiser et al. 2020). Implementing agricultural best management practices (BMPs) can enhance food security, create job opportunities, and support rural development (Anantha et al. 2021; Branca et al. 2013). Nitrogen offsets can further strengthen community engagement and education around sustainable land use, fostering collaboration among farmers, policymakers, and local organizations (Christensen et al. 2021).

Integrating environmental and social co-benefits into offset projects can also increase their economic value and attractiveness to investors and policymakers (Robertson et al. 2014). Although additional research is needed across regions to accurately quantify these benefits and support economic valuation (Robertson et al. 2014; von Hase and Cassin 2018), projects in high-co-benefit areas generally achieve greater returns by aligning with environmental and social regulations (Ellis et al. 2024), enhancing public and stakeholder acceptance (Fleming et al. 2019; Gough and Mander 2019), and attracting funding from conservation and carbon markets (Milne et al. 2024) (see Box 4).

Box 4 Economic, Social, and Environmental Challenges of Nitrogen Offsetting The multiple benefits generated by a single environmental offset are often interconnected and challenging to disentangle, complicating the accounting of credits when sold to different buyers (i.e., in cases of true credit stacking) (Robertson et al. 2014; von Hase and Cassin 2018). Unlike true credit stacking, selling credits from the same site as a package to a single buyer (i.e., credit bundling) facilitates the accounting of environmental offsets and helps minimize ecological losses. Credit bundling better upholds the principles of complete and symmetrical accounting by ensuring that key co-benefits are identified and that environmental gains and losses at impact sites are proportionally reflected at offset sites (Robertson et al. 2014; von Hase and Cassin 2018). Credit bundling also reduces concerns over “additionality” in credit stacking, thereby preventing “double dipping” (von Hase and Cassin^ 2018^).

Social and regulatory factors significantly influence nitrogen offset strategies, as public acceptance and policy alignment can determine their success or failure. Local communities and governments may oppose certain offset methods due to concerns about land use changes, potential livelihood disruptions, or environmental justice. For instance, while wastewater treatment wetlands effectively remove nitrogen, their feasibility in urban areas is limited by high land costs and competition with other development priorities (Matsuzaki et al. 2019; Sanon et al. 2012). Similarly, habitat restoration can improve water quality and deliver multiple environmental benefits, but requires careful planning to avoid conflicts with stakeholder interests, such as food production (Adame et al. 2019; Hagger et al. 2022; Miettinen et al. 2012; Smith et al. 2013). Regulatory frameworks may favor specific offset types that align with existing water quality standards or sustainability goals, limiting the range of viable offsetting options (Shortle 2013; Woodward and Kaiser 2002). Economic considerations further complicate decision-making, as financial incentives like subsidies or tax credits can make certain offsets more appealing, while high opportunity costs or long-term maintenance expenses may discourage investment (Heberling et al. 2010; Shortle 2013; Stephenson and Shabman 2017).

### Technical and Spatial Feasibility

The technical feasibility of nitrogen offsets depends on their implementability, scalability, and maintenance requirements, while their spatial feasibility involves ensuring that offsets are placed in locations where they provide the highest environmental impact. Spatially explicit analyses using Geographic Information Systems (GIS) and hydrological models can help identify the most suitable sites for offsets by integrating nitrogen removal potential, co-benefits, and geographic constraints (see section on *Inclusion of GIS into a Nitrogen Offsetting Framework*). By incorporating land-use data, hydrological flows, and soil characteristics, spatial modeling can prioritize areas where nitrogen offsets will be most effective, as these variables underpin watershed models used to simulate nitrogen transport (Hajati et al. 2020; Zhang et al. 2022). Additionally, feasibility assessments should account for regulatory constraints, land tenure issues, and potential conflicts with other land uses, as these socio-economic and governance factors strongly influence the implementation and success of offset programs (Bell-James et al. 2023; Saenz et al. 2013). Using predictive modeling to evaluate offset performance under different scenarios, such as climate change impacts and land-use changes, can further enhance the effectiveness of offset planning (Buonocore et al. 2021; Wade et al. 2022).

## A Framework To Identify, Select, And Implement Suitable Offset Types

**Fig. 7:**
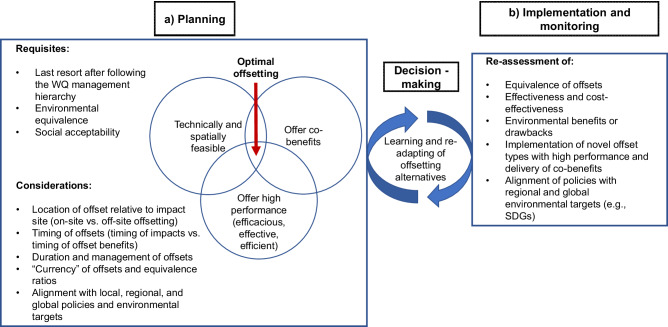
Key processes and considerations in water quality (WQ) offsetting as an adaptive process during the **a** planning and **b** implementation and monitoring phases. The WQ management hierarchy is defined in the Point Source Water Quality Offsets Policy (State of Queensland 2019)

Building on the considerations outlined above, we propose a framework to guide the identification, selection, and implementation of nitrogen offset strategies. This framework integrates scientific evidence on mitigation performance, co-benefits, and feasibility, alongside adaptive management supported by monitoring and spatial analysis. Its application involves two main phases. During the planning phase, the need for offsets is assessed, suitable locations are identified, and strategies are designed to meet regulatory and environmental objectives, based on the evaluation of options using scientific evidence, feasibility, and policy alignment to ensure measurable benefits (Fig. 7a). The implementation and monitoring phase involves executing offset activities, tracking their performance over time, and making necessary adjustments to maintain their effectiveness (Fig. 7b).

### **Requisites and Considerations During the Planning Phase (**Fig. 7a**)**

Nitrogen offsetting should be considered a last resort after all feasible measures to avoid, reduce, and mitigate nitrogen pollution at its source cannot be implemented (State of Queensland 2019). Offsets must also provide environmental equivalence, ensuring that the benefits they deliver match or exceed the impacts they are intended to compensate for (Bull et al. 2016; Maron et al. 2012), particularly in terms of water quality and ecosystem health (McMahon et al. 2024). Additionally, nitrogen offsetting schemes should be socially acceptable, meaning they are transparent, equitably distributed, and developed in consultation with local communities, traditional owners, and stakeholders to foster trust and long-term sustainability (Tupala et al. 2022; Varumo et al. 2023).

In addition to high nitrogen reduction performance, co-benefits, and technical and spatial feasibility, planning for optimal offsetting requires careful consideration of several key factors to ensure environmental integrity and long-term success. The location of offsets should be strategically chosen relative to the impact site to maximize water quality benefits within the affected catchment (Kiesecker et al. 2009; McMahon et al. 2024). Timing is also critical, as offsets should be implemented before or concurrently with the nitrogen-generating activity to prevent environmental degradation (Bull et al. 2015; Moilanen and Kotiaho 2018). Additionally, offsets must be managed and maintained appropriately to ensure their effectiveness over time (Haya et al. 2023). The currency of offsets and equivalence ratios should be designed to accurately reflect the scale and impact of nitrogen reductions, ensuring that offsets provide genuine and proportional environmental benefits (Lu et al. 2023). Finally, nitrogen offset programs should align with local, regional, and global targets for water quality improvement, biodiversity conservation, and climate resilience to contribute meaningfully to broader sustainability goals (Ellis et al. 2024; Kanter et al. 2020).

In countries where monitoring data are limited—such as those in Africa, Central and South America, Southeast Asia, and Pacific Island countries in Oceania—the framework can prioritize evidence from studies conducted under similar tropical and subtropical conditions, providing an initial basis for identifying suitable nitrogen reduction strategies. As previously discussed, studies reporting effectiveness (real-world performance) would be preferred, while results from studies reporting efficacy (controlled conditions) can be complemented with environmental modeling to estimate potential nitrogen reductions and considered with caution, allowing subsequent efficiency (cost-effectiveness) assessments to be adapted to local conditions. Importantly, the adaptive management component of the framework would allow offset strategies to be implemented using the best available information and progressively refined as monitoring data and local evidence become available (see the following section).

### **Continuous Improvement: Learning from Implementation and Monitoring (**Fig. 7b**)**

Enhancing the environmental performance of nitrogen offset strategies requires the continuous integration of lessons from past and ongoing projects. This involves reassessing the environmental equivalence of implemented offsets to verify that predicted nitrogen reductions align with actual outcomes. It also requires incorporating data on effectiveness, cost-effectiveness, benefits, trade-offs, and drawbacks into future planning. Promising, high-performance offsets and novel approaches with added co-benefits can further strengthen environmental outcomes. Finally, regular reassessment helps ensure nitrogen offsets remain aligned with evolving policies and broader goals for water quality, biodiversity, and climate resilience.

## Inclusion of GIS Into A Nitrogen Offsetting Framework

**Fig. 8:**
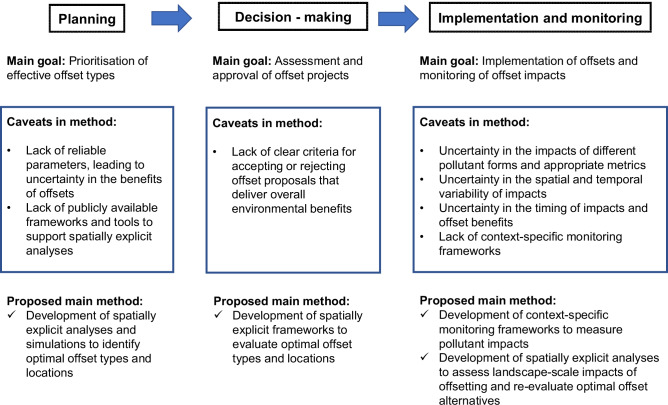
Caveats and proposed solutions for achieving environmental benefits during the planning, decision-making, and implementation of offsets

During the offsetting planning phase, unreliable or outdated baseline data (Bull et al. 2013) can hinder the selection of appropriate sites and offset types, potentially leading to poorly designed offsets that fail to deliver the proposed environmental benefits. Developing a spatially explicit and flexible prioritization framework for nitrogen offsetting, designed to maximize the efficiency of nitrogen reduction while also delivering co-benefits could help in planning nitrogen offsetting (Fig. 8). Such spatial tools can support the integration of scientific evidence on mitigation performance, the identification of suitable offset locations and types, and adaptive management through improved monitoring and data integration. Rather than treating nitrogen offsets as isolated interventions, the GIS framework should aim for incremental environmental improvement, ensuring offsets go beyond business-as-usual improvements (Morrison-Saunders and Sánchez 2024). Flexibility in this prioritization framework can facilitate the inclusion of new scientific knowledge, evolving legislation, updated geographic information with a higher spatial and temporal resolution, and changes to development and land-use baselines to account for additionality and, ideally, leakage effects (i.e., preventing displacement of impacts to other areas) (Filewod and McCarney 2023; Moilanen and Laitila 2016). Proponents of development activities could integrate this prioritization framework into their approval processes, ensuring that offset proposals align with broader conservation and sustainability goals. Regulators and assessors could also leverage the framework to evaluate the effectiveness of nitrogen offsets once implemented, promoting greater accountability and consistency in offset performance. Importantly, the framework should be adaptable, allowing for the incorporation of new scientific knowledge, evolving legislation, and improved geographic data with higher spatial and temporal resolution.

## Conclusions

Acknowledging how nitrogen reduction differs across water quality improvement methods is critical for identifying and selecting appropriate nitrogen offsets. By integrating this evidence into a structured decision-support framework, our analysis showed that treatment wetlands performed strongly for dissolved inorganic nitrogen (DIN) reduction, while bioreactors were particularly effective for TN reduction. These findings provide an important basis for identifying suitable nitrogen offset types in tropical and subtropical regions, including the Great Barrier Reef Catchment Area. However, caution is needed when applying these results to specific sites, as performance is highly location-dependent and the available literature remains limited. Expanding research on implemented offsets and water quality improvement measures—particularly in terms of effectiveness and efficiency—is therefore essential, especially for underrepresented approaches such as habitat restoration based on riparian buffers, which also provide important co-benefits for biodiversity conservation and carbon storage.

The long-term success of nitrogen offsets will depend on integrating scientific evidence on mitigation performance with the careful selection of offset types and adaptive management supported by monitoring and spatial planning tools. Spatially explicit approaches can help identify optimal offset locations and strategies while incorporating key principles such as environmental equivalence, additionality, and leakage. The framework presented here provides a structured and flexible approach to support these decisions, enabling developers, regulators, and land and water managers to design, evaluate, and prioritize nitrogen offset strategies that maximize environmental benefits and co-benefits across catchments. By supporting the integration of new data, evolving policies, and improved analytical tools, this framework contributes to strengthening the scientific basis and practical implementation of nitrogen offsetting. In doing so, it can enhance the credibility and effectiveness of emerging offset markets and support the development of more adaptive, transparent, and sustainable water quality management strategies.

## Supplementary information

Supplementary information

## Data Availability

All data supporting the findings of this study are available within the paper and its Supplementary Information.
